# Blood-Rich Enhancement in Ultrasonography Predicts Worse Prognosis in Patients With Papillary Thyroid Cancer

**DOI:** 10.3389/fonc.2020.546378

**Published:** 2021-01-08

**Authors:** Luying Gao, Xuehua Xi, Qiong Gao, Jiajia Tang, Xiao Yang, Shenling Zhu, Ruina Zhao, Xingjian Lai, Xiaoyan Zhang, Bo Zhang, Yuxin Jiang

**Affiliations:** ^1^ Department of Ultrasound, Peking Union Medical College Hospital, Chinese Academy of Medical Sciences & Peking Union Medical College, Beijing, China; ^2^ Department of Ultrasound, China-Japan Friendship Hospital, Beijing, China

**Keywords:** thyroid nodule, papillary thyroid cancer, ultrasound, contrast-enhanced ultrasound, prognosis

## Abstract

Contrast-enhanced ultrasound (CEUS) can be used to evaluate microcirculation in cancers, which in turn is associated with the biologic features and ultimately patient prognosis. We conducted a retrospective analysis to examine potential association between CEUS parameters and prognosis in patients with papillary thyroid cancer (PTC). The analysis included 306 patients who underwent CEUS prior to thyroidectomy at our center during a period from 2012 to 2019. Subjects with excellent response (ER) were compared to the non-ER group (including indeterminate response, biochemical incomplete response and structural incomplete response). During the median follow-up of 34 months, ER was observed in 195 (63.7%) subjects. The remaining 111 (36.3%) patients developed non-ER events, with distant metastasis in five (1.6%) cases. In a multivariate COX regression, non-ER event was associated with the male sex (OR = 1.83, 95%CI: 1.21–2.76) and blood-rich enhancement in CEUS (OR = 1.69, 95%CI: 1.04–2.75). Based on this finding, we developed a predictive model: high risk for developing non-ER events was defined as having both risk factors; low risk was defined as having none or only one risk. In receiver operating characteristic (ROC) analysis, the area under the curve was 0.59 (95%CI: 0.52–0.66). The sensitivity and specificity were 17.1 and 95.4%, respectively. The positive and negative predictive values were 67.9 and 66.9%, respectively. In conclusion, blood-rich enhancement in CEUS is associated with non-ER events after thyroidectomy in patients with PTC.

## Introduction

Papillary thyroid cancer (PTC) accounts for 85% of differentiated thyroid cancers (1). After surgical resection, recurrence rate is up to 30% (2–4), with distant metastasis at about 1% (5). Surgery can improve patient prognosis, but may lead to a number of complications, including hypoparathyroidism and recurrent laryngeal nerve injury (6). In several recent studies, long-term benefits were not observed only in patients with high-risk and not in patients with low-risk PTC (7, 8). Factors that are known to be associated with poor prognosis include older age (>55 years), male sex, larger tumor size, extra-thyroid extension, and distant metastasis (9, 10).

Ultrasonography is the preferred method for pre- and post-operative follow-up in thyroid cancer patients (1). In PTC patients, regional and distant metastases could occur through either newly formed or preexisting blood vessels (11). In comparison to the limited information about vascularity available from conventional ultrasonography, contrast-enhanced ultrasonography (CEUS) could be used to evaluate microvascular density (12). A previous study suggested an association between hyper-iso enhancement of CEUS and central lymph node metastasis in PTC (13). In comparison to conventional ultrasound, CEUS also has higher sensitivity and specificity for detecting extrathyroidal extension (14). CEUS has also been used to predict the prognosis in patients with other diseases, including hepatic cirrhosis and pancreatic carcinoma (15, 16).

We conducted a retrospective analysis to examine whether CEUS parameters, particularly enhancement grade and pattern, differ significantly between PTC patients with good vs. poor prognosis.

## Materials and Methods

### Study Cohort

In this retrospective analysis, we screened all adult patients (18 years of age or older) undergoing both conventional ultrasound and CEUS prior to total or near-total thyroidectomy during a period from January 2012 and April 2019 at the Peking Union Medical College Hospital. The study was approved by the hospital Ethics Committee, and the number of Ethics Committee approval is JS-1671. For inclusion in the analysis, the diagnosis of PTC (either classical or follicular) must be based on pathology and the diameter of the largest nodule must be >5 mm. Follow-up was conducted periodically and consisted of conventional ultrasound and plasma thyroid-stimulating hormone (TSH), thyroglobulin (Tg), and anti-Tg antibody. In the cases with suspected recurrence, patients underwent whole-body 131-iodine magnetic resonance imaging (MRI), computed tomography (CT), or 18-fluorodeoxyglucose positron emission tomography (PET).

Treatment response was categorized based on the 2015 American Thyroid Association (ATA) guidelines to: excellent response (ER), indeterminate response (IDR), biochemical incomplete response (BIR), and structural incomplete response (SIR). ER was defined as no clinical, biochemical, or structural evidence of disease (suppressed Tg <0.2 ng/ml or TSH-stimulated Tg <1 ng/ml). IDR was defined as biochemical or structural findings that could not be classified as either benign or malignant (weak iodine uptake in thyroid bed; non-stimulated Tg detectable but <1 ng/ml; stimulated Tg detectable but <10 ng/ml, or stable/declining Tg antibody in the absence of structural or functional disease). BIR was defined as abnormal Tg in the absence of localized disease (negative imaging and suppressed Tg >1 ng/ml or stimulated Tg >10 ng/ml or rising Tg antibody). SIR was defined as persistent or newly identified loco-regional or distant metastasis (structural or functional evidence of disease with any Tg level and Tg antibody status) (1). In our analysis, IDR, BIR, and SIR were combined into a non-ER group.

### Ultrasound Examination

Ultrasound examination was conducted using a 5-12-MHz probe (iU22; Philips Medical Systems, Bothell, WA, USA) in all subjects. For patients with two or more thyroid nodules, only the one with largest diameter was included in the analysis. The evaluation was conducted based on the 2015 ATA guidelines (1). High suspicion was defined as solid hypoechoic nodule or solid hypoechoic component of a partially cystic nodule with one or more of the following characteristics: irregular margin (infiltrative, micro-lobulated), microcalcification, taller-than-wide shape, disrupted rim calcification with small extrusive soft tissue component, or evidence of extrathyroidal extension. Intermediate suspicion was defined as a hypoechoic solid nodule without any of the above-mentioned features. Low suspicion was defined as and isoechoic or hyperechoic solid nodule or partially cystic nodule with eccentric solid areas without any of the above-mentioned features. Very-low suspicion was defined as spongiform or partially cystic nodules without any of the above-mentioned features. Cystic nodules with no solid component were considered benign. Vascularity was classified into three types based on color Doppler imaging: I: absence of intranodular or perinodular flow; II: presence of perinodular and/or slight intranodular flow; III: presence of marked intranodular and slight perinodular flow (17).

The contrast medium for CEUS was SonoVue (Bracco Imaging, Milan, Italy). Enhancement was graded based on contrast intensity at the peak (relative to that of the parenchyma) to either low or blood-rich enhancement. Blood-rich enhancement was defined that the contrast intensity was higher than that of parenchyma according to signal intensity of bubble at the peak time, and low enhancement was defined that the contrast intensity was equal to or lower than that of the parenchyma. Enhancement pattern was categorized as homogeneous, heterogeneous or ring-enhancement. Elastography images were generated after completing conventional ultrasound by the same radiologists, and scored using a five-point scale, adding elastography score (ESS) 0 to the version of Asteria criteria: a score of 0 was defined as cystic and predominantly cystic nodules with red, blue, or red and green, or blue and green color. 1: homogeneously green; 2: predominantly green; 3: predominantly blue; and 4: completely blue (18).

The images of conventional ultrasound, elastography, and CEUS were reviewed by two experienced radiologists who were blinded to all other information. Discrepancies were resolved through discussion.

### Statistical Analysis

Categorical variables are presented as number and frequency, and analyzed using the χ2 test. A Cox proportional hazards model was used to identify the factors associated with poor prognosis (non-ER responses). Survival curves were plotted using the Kaplan–Meier method, and analyzed using a log-rank test. A receiver operating characteristic (ROC) analysis was conducted to analyze the sensitivity, specificity, positive predictive value (PPV), negative predictive value (NPV) and accuracy. Inter-observer variability was examined using the kappa value. All statistical analyses were conducted using SPSS software version 19.0 (IBM, Armonk, NY, USA). Differences with p <0.05 were considered statistically significant.

## Results

**Figure 1:**
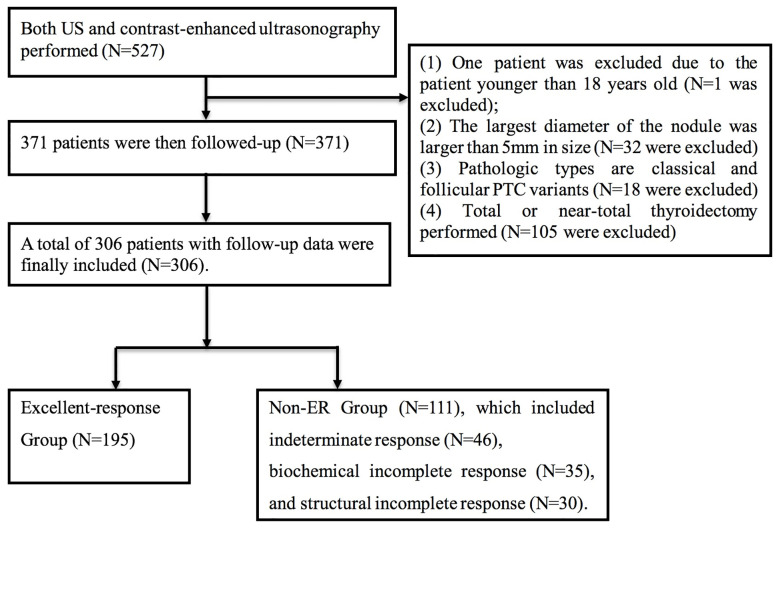
Flowchart of patient selection and inclusion in the study.

**Figure 2:**
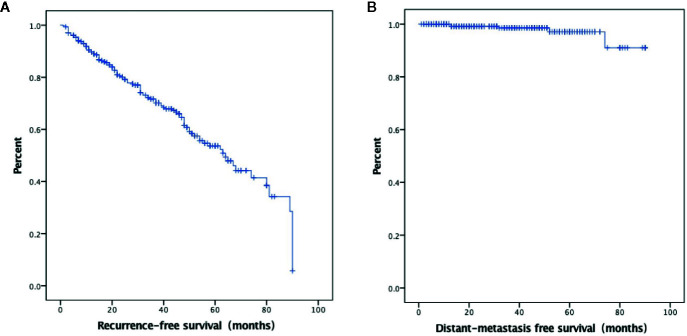
Kaplan–Meier curves showing excellent response survival **(A)** and distant metastasis-free survival **(B)**.

A total of 527 patients were screened. The final analysis included 306 patients with follow-up data (mean age: 42.7 ± 11.1 years; 77 men) (**Figure 1**). The median follow-up was 34.3 months (range: 1–90 months). At the time of thyroidectomy, none of the patients had distant metastasis. Treatment responses included ER in 195 (63.7%) patients, IDR in 46 (15.0%) patients, BIR in 35 (11.4%) patients, and SIR in 30 (9.8%) patients (**Figure 2A**). The ER rate was 70.1% at three years and 52.3% at 5 years. Five patients developed distant metastases (four to the lungs and one to the liver) (**Figure 2B**). Inter-observer agreement for evaluating the enhancement grade was fair (kappa = 0.92).

In the analysis that included only the 77 men, treatment responses included ER in 45 (58.4%) patients, IDR in eight (10.4%) patients, BIR in 11 (14.3%) patients and SIR in 13 (16.9%) patients. Distant metastases occurred in two (2.6%) patients. In the subgroup analysis that included only the 229 women, treatment responses included ER in 150 (65.5%) patients, IDR in 38 (16.6%) patients, BIR in 24 (10.5%) patients and SIR in 17 (7.4%) patients. Distant metastases occurred in three (1.3%) patients.

Demographic, clinicopathologic, and ultrasound features in the ER group *vs*. non-ER group are summarized in **Table 1**. Briefly, the two groups did not differ in age. Men accounted for higher percentage in the non-ER group (32.4 *vs*. 21.0% in the ER group, p = 0.03). In comparison to the ER group, the non-ER group also had higher rate of blood-rich enhancement (48.6 *vs*. 30.8%, p = 0.003). The two groups did not differ significantly in any other ultrasound features, including nodule diameter, margin, microcalcification, halo, internal component, echogenicity, ATA risk stratification, color doppler flow imaging type, elastography, enhanced pattern, relative exit time and relative microbubble arrival time. Pathological features, including capsule invasion, lymph node metastasis on pathology and large-volume lymph node metastasis on pathology, did not differ between the two groups.

**Table 1:** Demographic and clinicopathologic characteristics in patients with excellent response (ER) vs. non-ER (including indeterminate, biochemical incomplete, and structural incomplete responses).

Characteristic	ER (n = 195)	Non-ER (n = 111)	P value
Age, years (mean ± SD)	42.3 ± 11.4	43.4 ± 10.5	
>55 years	33 (16.9)	14 (12.6)	0.20
Male sex	41 (21.0)	36 (32.4)	**0.03**
LN dissection			0.23
Central	53 (27.2)	23 (20.7)	
Central and lateral	28 (14.4)	23 (20.7)	
Ultrasound features			
Diameter >1 cm	90 (46.2)	48 (43.2)	0.13
Irregular margin	179 (87.4)	97 (91.8)	0.15
Microcalcification	116 (59.5)	60 (54.1)	0.21
Halo	191 (97.9)	106 (95.5)	0.19
Internal component			0.20
Solid	182 (93.3%)	107 (97.4%)	
Solid-cystic	13 (6.7%)	4 (3.6%)	
Echogenicity			0.40
Hyper	189 (96.9%)	109 (98.2%)	
Hyper-iso	6 (3.1%)	2 (1.8%)	
ATA risk stratification			0.33
High suspicion	183 (93.8%)	101 (91.0%)	
Intermediate - very low suspicion	12 (6.2%)	10 (9.0%)	
Vascularity			0.11
Type I	13 (6.7)	12 (10.8)	
Type II	122 (62.6)	76 (68.5)	
Type III	60 (30.8)	23 (20.7)	
Elasticity score			0.54
0–2	24 (12.3)	14 (12.6)	
3/4	171 (87.7)	97 (87.4)	
CEUS features			
Heterogeneous enhancement pattern	177 (90.8)	94 (84.7)	0.079
Enhancement grade			**0.003**
Blood-rich	60 (30.8)	54 (48.6)	
Low	135 (69.2)	67 (51.4)	
Relative arrival time of microbubble			0.15
Earlier or meantime	78 (40.0)	52 (46.8)	
Later	117 (60.0)	59 (53.2)	
Relative exit time of microbubble			0.22
Earlier or meantime	64 (32.8)	42 (37.8)	
Later	131 (67.8)	69 (62.2)	
Suspected LN on preoperative ultrasound	55 (28.2)	30 (27.0)	0.47
Pathological features			
Capsule invasion	10 (5.2)	6 (5.5)	0.51
LN metastasis	82 (42.3)	48 (43.2)	0.53
Large-volume LN metastasis	30 (15.4)	18 (16.2)	0.48

Values are expressed as n (%) unless indicated. Number reported in bold are statistically significant.

SD, standard deviation; LN, lymph node; ATA, American Thyroid Association.

**Figure 3:**
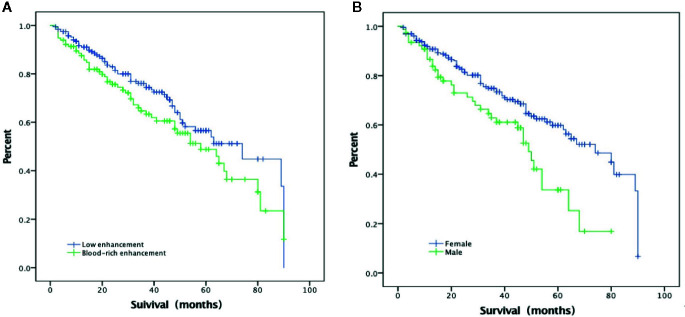
Excellent response based on **(A)** blood-rich enhancement in CEUS and **(B)** sex.

**Figure 4:**
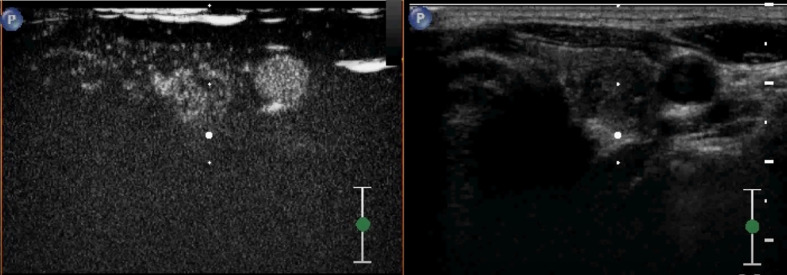
A case of a 38-year-old woman with incidentally detected thyroid nodule. CEUS revealed a solid hypoechoic nodule with blood-rich enhancement in the left thyroid lobe. This patient developed cervical lymph node recurrence 25 months after the surgery.

In the multivariate Cox regression, poor prognosis (non-ER) was associated with blood-rich enhancement (odds ratio, OR, 1.69, 95%CI 1.04–2.75, p = 0.035) and the male sex (OR=1.83, 95%CI 1.21–2.76, p = 0.004) (**Table 2** and **Figures 3** and **4**). A prognostic model was built based on these findings: the presence of both factors was classified as high risk; the presence of one or none was classified as low risk. In the ROC analysis, the area under the curve (AUC) of this model was 0.59 (95%CI 0.52–0.65, p = 0.012) for predicting non-ER. The model had 17.1% sensitivity and 95.4% specificity. The PPV was 67.9. The NPV was 66.9%. The overall accuracy was 67.0%.

**Table 2:** Multivariate analysis of the risk factors for poor prognosis (non-ER).

	β	SE	Wald	P-value	OR	95% CI
Male	0.603	0.21	8.212	**0.004**	1.827	1.21–2.76
Age (>55 years)	−0.106	0.295	0.13	0.718	0.899	0.504–1.603
Size (>1 cm)	−0.289	0.207	1.955	0.162	0.749	0.499–1.123
Solid components	0.15	0.533	0.079	0.779	1.161	0.409–3.298
Halo	−1.002	0.51	3.851	0.050	0.367	0.135–0.999
Later relative arrival time of microbubble	0.074	0.246	0.09	0.764	1.077	0.665–1.744
Blood-rich enhancement	0.524	0.249	4.423	**0.035**	1.688	1.036–2.75
Heterogeneous enhancement pattern	0.101	0.303	0.111	0.739	1.106	0.611–2.002
Margin	0.054	0.321	0.028	0.867	1.055	0.562–1.981

Number reported in bold are statistically significant.

OR, odds ratio; CI, confidence interval.

## Discussion

In the current study, poor prognosis (as defined by non-ER) was independently associated with the male sex and blood-rich enhancement on preoperative CEUS in adult PTC patients with >5 mm nodules. A prognostic model based on these two factors yielded 67.0% accuracy in predicting non-ER. This model has poor sensitivity (17.1%) but high specificity (95.4%). The PPV and NPV were 67.9 and 66.9%, respectively. Considering the high survival rate in PTC patients with recurrence, we believe that this model could provide valuable information in decision-making in clinical practice.

Regional and distant metastases of PTC could occur through either newly formed or preexisting blood vessels (11). Over-expression of vascular growth factor A (VEGF-A) and high microvascular density have been associated with poor prognosis in PTC (19). CEUS could be used to evaluate angiogenesis *in vivo* (12), and to assess microvascular density in PTC (20, 21). In the current study, the non-ER group had higher rate of blood-rich enhancement. This finding is consistent with several previous studies in which the intensity of enhancement at peak time is associated with positive lymph node status (22, 23), and adds support to the use of CEUS in identifying highly aggressive PTC (24).

Estimated recurrence rate of PTC varies widely from 1.4 to 29.0% (25). Poor prognosis has been associated with a variety of factors, including male sex, younger age, larger tumor size, cervical lymph node metastasis and distant metastasis (9, 10). Previous studies have associated male sex with more advanced disease, lower disease-specific survival and higher rate of recurrence (26, 27). In a study of 15,698 cases of thyroid cancer, sex was found to be a significant prognostic factor, with men having lower disease-specific survival and higher rate of recurrence (28). In the current study, men had higher risk for developing non-ER events after thyroidectomy (OR = 1.83, 95%CI 1.21–2.76). The role of sex in PTC patients remain controversial. For example, a previous study suggested that expression of estrogen receptor in thyroid cancer cells could promote cell proliferation (29), and at least one study failed to find association between male sex and PTC prognosis (30).

Our study has several limitations. First, selection bias is apparent: only patients who underwent thyroidectomy were included in the analysis. Second, the follow-up duration varied widely across the study subjects (from 1 to 90 months) despite of the 34.3-month median. Thirdly, the retrospective nature of the study could have introduced many poorly controlled confounding factors (both known and unknown). Mostly importantly, the prognostic model based on the male sex and blood-rich enhancement has high specificity but very low sensitivity. Future studies with large sample size, and perhaps more careful and meaningful prognostic measures, are needed to verify our findings.

## Conclusions

In PTC patients undergoing total or near-total thyroidectomy, non-ER events are associated with the male sex and blood-rich enhancement in CEUS. With further improvement, these findings may be useful in identifying patients at higher risk for non-ER events.

## Data Availability Statement

The raw data supporting the conclusions of this article will be made available by the authors, without undue reservation.

## Ethics Statement

The studies involving human participants were reviewed and approved by The ethics committee of the principal investigator’s hospital (Peking Union Medical College Hospital). The patients/participants provided their written informed consent to participate in this study. Written informed consent was obtained from the individual(s) for the publication of any potentially identifiable images or data included in this article.

## Author Contributions

BZ conceived the general idea and designed the study. LG participated in data collection and analysis, and manuscript preparation. YJ and XX contributed to the study design and participated in manuscript preparation. JT, QG, XY, SZ, RZ, XL, and XZ participated in data processing and image analysis. All authors contributed to the article and approved the submitted version.

## Funding

This study was supported by a grant from the International Science and Technology Cooperation Program of China (no. 2015DFA30440), the National Natural Science Foundation of China (no. 81541131), the Capital Health Research and Development of Special (no. CD 2016-2-40110) and the Spatial-Temporal Mapping Analysis on Chinese Cancer Burden (2018-I2M-3-003).

## Conflict of Interest

The authors declare that the research was conducted in the absence of any commercial or financial relationships that could be construed as a potential conflict of interest.
